# Contamination of Soil, Water, Fresh Produce, and Bivalve Mollusks with *Toxoplasma gondii* Oocysts: A Systematic Review

**DOI:** 10.3390/microorganisms10030517

**Published:** 2022-02-27

**Authors:** Nadia María López Ureña, Umer Chaudhry, Rafael Calero Bernal, Santiago Cano Alsua, Davide Messina, Francisco Evangelista, Martha Betson, Marco Lalle, Pikka Jokelainen, Luis Miguel Ortega Mora, Gema Álvarez García

**Affiliations:** 1 SALUVET Research Group, Animal Health Department, Veterinary Faculty, Complutense University of Madrid, 28040 Madrid, Spain; nadiamlo@ucm.es (N.M.L.U.); r.calero@ucm.es (R.C.B.); luis.ortega@ucm.es (L.M.O.M.); 2 Veterinary Epidemiology and Public Health Department, School of Veterinary Medicine, University of Surrey, Guildford GU2 7XH, UK; u.chaudhry@surrey.ac.uk (U.C.); or davide.messina1@nottingham.ac.uk (D.M.); f.evangelista@surrey.ac.uk (F.E.); m.betson@surrey.ac.uk (M.B.); 3 Computing Services, Research Support Center, Complutense University of Madrid, 28040 Madrid, Spain; scano@ucm.es; 4 Division of Veterinary Clinical Science, School of Veterinary Medicine and Science, University of Nottingham, Sutton Bonington, Loughborough LE12 5RD, UK; 5 Unit of Foodborne and Neglected Parasitic Diseases, Department of Infectious Diseases, Istituto Superiore di Sanità, 00161 Roma, Italy; marco.lalle@iss.it; 6 Department of Bacteria, Parasites and Fungi, Infectious Disease Preparedness, Statens Serum Institute, University of Copenhagen, 2300 Copenhagen, Denmark; pijo@ssi.dk

**Keywords:** *Toxoplasma gondii*, oocysts, environment, soil, water, fresh produce, fruit, bivalve mollusk, sampling strategy, methodology

## Abstract

*Toxoplasma gondii* is a major foodborne pathogen capable of infecting all warm-blooded animals, including humans. Although oocyst-associated toxoplasmosis outbreaks have been documented, the relevance of the environmental transmission route remains poorly investigated. Thus, we carried out an extensive systematic review on *T. gondii* oocyst contamination of soil, water, fresh produce, and mollusk bivalves, following the PRISMA guidelines. Studies published up to the end of 2020 were searched for in public databases and screened. The reference sections of the selected articles were examined to identify additional studies. A total of 102 out of 3201 articles were selected: 34 articles focused on soil, 40 focused on water, 23 focused on fresh produce (vegetables/fruits), and 21 focused on bivalve mollusks. *Toxoplasma gondii* oocysts were found in all matrices worldwide, with detection rates ranging from 0.09% (1/1109) to 100% (8/8) using bioassay or PCR-based detection methods. There was a high heterogeneity (I^2^ = 98.9%), which was influenced by both the sampling strategy (e.g., sampling site and sample type, sample composition, sample origin, season, number of samples, cat presence) and methodology (recovery and detection methods). Harmonized approaches are needed for the detection of *T. gondii* in different environmental matrices in order to obtain robust and comparable results.

## 1. Introduction

Toxoplasmosis is one of the most important opportunistic parasitic diseases affecting humans and animals worldwide and is caused by the obligate intracellular protist *Toxoplasma gondii*. Clinical manifestations associated with toxoplasmosis are various, and they include ocular disease [1,2], pneumonia [3,4], and encephalitis in immunocompromised patients [1,5]. *Toxoplasma gondii* infection can also cause spontaneous abortion, congenital malformations, and stillbirth in both humans and animals [6,7].

Domestic and wild felids are the specific definitive hosts of *T. gondii*, whereas warm-blooded vertebrates, including humans, are intermediate hosts [8]. Up to 70% of the cat population is infected with *T. gondii* [9], and the infected cats can shed millions of oocysts in their feces. The subsequent development of sporulated oocysts in the environment depends on temperature and humidity [10,11].

Humans, as well as animals, can become infected with *T. gondii* through the consumption of raw or undercooked meat of infected animals harboring the tissue-dwelling stages of the parasite (bradyzoites contained within tissue cysts) [12] as well as via congenital transmission and blood transfusion by the active replicative stages of the parasite (tachyzoites) [10]. Another important route of human and animal infection is through the ingestion of sporulated *T. gondii* oocysts present in the environment, contaminating soil, water, and feed and food, including fresh produce and seafood [13]. According to a systematic review of studies carried out up to March 2018, 44.1% (15/34) of documented worldwide outbreaks were oocyst-related [14].

Soil contamination is a significant source of human infection, with soil of public parks, schools, gardens, and farms considered particularly important. Oocysts can be distributed within the soil by arthropods, earthworms, wind, and rain [7], and the sporulated oocysts are highly resistant and can persist infective in soil for up to two years [11].

Waterborne infections associated with *T. gondii* oocysts are nowadays considered increasingly significant due to evidence of large-scale outbreaks [7,13]. Water in irrigation systems, rivers, lakes, beaches, and coasts, as well as wastewater and groundwater can be contaminated with the environmentally resistant oocysts. Moreover, oocysts can survive various inactivation procedures using chemical reagents, including sodium hypochlorite and chlorine [15,16]. Oocysts remain viable in water for 18 months at 4 °C after exposure to 2% sulfuric acid [7,17], for 15 and 54 months at 20–25 °C and 4 °C in fresh water, respectively, and around 6 months in artificial seawater (15 ppt) at the same temperatures [18].

In recent years, *T. gondii* infection cases linked to fresh vegetable consumption have been on the increase [14]. Oocyst contamination of fresh vegetables may occur through cultivation in contaminated soil or using contaminated water for irrigation or washing. As testing for parasite contamination in fresh produce is neither regulated nor mandatory, the increased popularity of consumption of raw and ready-to-eat vegetables may pose a new potential risk for consumers who could be accidentally exposed to oocysts, since most post-harvest processing measures do not guarantee the complete removal of oocysts or their effective inactivation [16,19].

*Toxoplasma gondii* oocysts can also enter the marine environment through improper disposal of sewage, inefficient treatment plants, water discharge, and water runoff [20], and they can cause infections in marine animals and the contamination of marine fauna [21,22]. Consistently, oocysts have been detected in wild and commercial bivalve mollusks in several countries. Bivalves continuously filter large volumes of water and concentrate microorganisms [23]. They can retain viable *T. gondii* oocysts for 85 days following uptake [24]. Thus, they are considered good biological indicators of parasitic contamination of aquatic environments and could pose another risk for consumers when consumed undercooked or raw [18,25].

Environmental contamination with *T. gondii* oocysts is understudied and likely underestimated, which is partly due to the lack of suitable harmonized sampling approaches and detection methods. Studies on cat feces or susceptible intermediate hosts have been used as a substitute to predict the level of environmental contamination [26,27], but they may have inadequate power to accurately assess contamination. Due to limited baseline data on oocyst occurrence in environmental samples, accurate estimation of the contamination in the environment requires large sample sizes and sample volumes, which may contain small quantities of oocysts of different ages [7]. Limitations in oocyst recovery and detection methods, in combination with various sampling strategies, have made it difficult to ascertain the contribution of environmental contamination with *T. gondii* oocysts to human infections. Indirect methods for discriminating between infections caused by oocysts vs. tissue-dwelling stages of *T. gondii* have been developed but have not been widely applied [28].

Another important challenge to full evaluation of the relevance of *T. gondii* oocyst infection route is the assessment and quantification of oocyst viability and therefore infectivity for humans and animals. So far, the only reliable method is based on mouse bioassay, i.e., experimental administration of oocysts to mice and detection of infection in tissues [8], although new approaches based on molecular methods have been proposed and are under evaluation for their applicability [29,30,31].

To date, reviews on *T. gondii* environmental contamination of one [18,32] or more matrices [7], and systematic reviews covering one matrix exist [9,33,34,35]; however, they mainly focused on detection rates or analytical methods. Thus, this article aims to provide a more complete, comprehensive systematic review of the existing literature on environmental contamination with *T. gondii* oocysts, including available data on sampling strategies, and identifying relevant knowledge gaps and limitations in relation to sampling strategies and methods for the recovery and detection of *T. gondii* oocysts in soil, water, fresh produce, and bivalve mollusks. Finally, based on the observations, recommendations are suggested for future studies on environmental contamination with *T. gondii* oocysts.

## 2. Materials and Methods

### 2.1. Literature Search and Eligibility Criteria

A systematic review of the prevalence of *T. gondii* oocysts in soil, water, fresh produce (vegetables and fruits), and bivalve mollusks worldwide was performed; all papers published, with no restriction on language, until the end of 2020 were included, following the Preferred Reporting Items for Systematic Reviews and Meta-Analyses (PRISMA) [36].

The databases used were PubMed, Web of Science, and Scopus. In all cases, a combination of three search terms was employed and included “*Toxoplasma*” and “oocysts” or “oocyst” and “vegetables” or “fruits” or “ready to eat” or “salads” or “greenery” or “water” or “soil” or “bivalves” or “mussels” or “clams” or “oysters” or “abalone”. In view of the diversity of terms yielding eligible studies, an additional search was performed using related terms such as “food” or “products” or “otters”. Additionally, the bibliographies of the selected articles were screened to identify studies to include (Supplementary Table S1).

The articles were selected using the following inclusion criteria: worldwide studies reporting direct detection of *T. gondii* oocysts in one of the matrices of interest (soil, water, fresh produce, and bivalves) with full text available. Exclusion criteria were the following: methodological studies aiming only to the development or improvement of oocyst recovery or detection methods (i.e., using artificial spiking of matrices), studies performed on other matrices, studies without available full text, studies published after 2020, and duplicates.

### 2.2. Selection Process and Data Extraction

Three investigators carried out the initial screening focusing on title and abstract, and based on this, eligible articles were preselected and subjected to an in-depth review to confirm if they met the selection criteria. Subsequently, data extraction was carried out by two co-authors, and a third co-author resolved discrepancies.

For data extraction, one data sheet per matrix was created in Microsoft Excel 2013. For all matrices, the data sheet included sample type/details and origin, sampling year and season, period, country and continent, samples by categories (*n*), total number of samples, sample units, presence of cats in the sampling area, association with human *T. gondii* infection or toxoplasmosis (outbreaks or sporadic cases), positive samples by categories (number and percentage), total number of positive samples (number and percentage), sample collection and preparation, oocyst recovery and detection methods, DNA extraction method and molecular markers used, oocyst quantification (mean, median, and range), analytical sensitivity (Se), additional molecular methods used, source of information, journal subject area, and other parasites investigated (Supplementary Tables S2–S5).

Specific columns were also included in the spreadsheets according to the type of matrix. For soil, there were columns related to sampling site, sample size, and depth of sample collection (cm). Columns related to the type of aquifer, the uses, and the treatment received were added for water, and matrix composition, product type (organic, conventional or both), and product presentation (bulk, packaged, or ready-to-eat (RTE)) for fresh produce. Finally, for bivalves, columns related to sampled species, sampling site, specimen length (cm), depth of collection (cm), and type of tissue or material analyzed were added.

When a study analyzed two or more matrices, data were extracted and considered separately for each matrix. The data extracted were limited to the information provided in the articles.

### 2.3. Data Analyses

Several studies reported oocyst detection by light microscopy or direct visualization of *T. gondii* oocysts by autofluorescence using an epifluorescent microscope as the only or initial screening method. However, since these techniques cannot prove the identity of *T. gondii* oocysts due to their shape and size similarity with several genera and species of the Sarcocystidae family, and because oocyst wall autofluorescence is not an exclusive feature of *T. gondii*, data based on microscopy findings were extracted and included in Supplementary Tables S2–S5, but they were not considered for data analyses. Accordingly, only data based on molecular and bioassay methods were included in the Results and Discussion sections. Moreover, only data from individual experimental samples were included in the analysis, not data from pooled samples. Regarding fresh produce, it was not always clear whether pooled samples were analyzed. Thus, if the mass of the sample analyzed was greater than the sample unit mass, it was considered to be a pool and was consequently excluded (e.g., sample units of 3600 g of lettuce [37] or 1000 g of strawberries [38]).

For the evaluation of heterogeneity and pooled estimates, detection rates reported in each study were combined per matrix (soil, water, fresh produce, bivalve mollusks), using STATA 15.0 software (StataCorp, Bryan, TX, USA) and a restricted maximum likelihood method with a random effects model. A Forest plot was created for easy data deviation within matrix type (Supplementary Figures S1–S4). The inverse variance index (I^2^) was used to quantify heterogeneity [39,40]. In addition, study bias and heterogeneities at the study level were calculated by Egger’s test, funnel plots (Supplementary Figure S5), and Cochran’s Q test, respectively [41].

## 3. Results and Discussion

### 3.1. Literature Search and Article Selection

**Figure 1:**
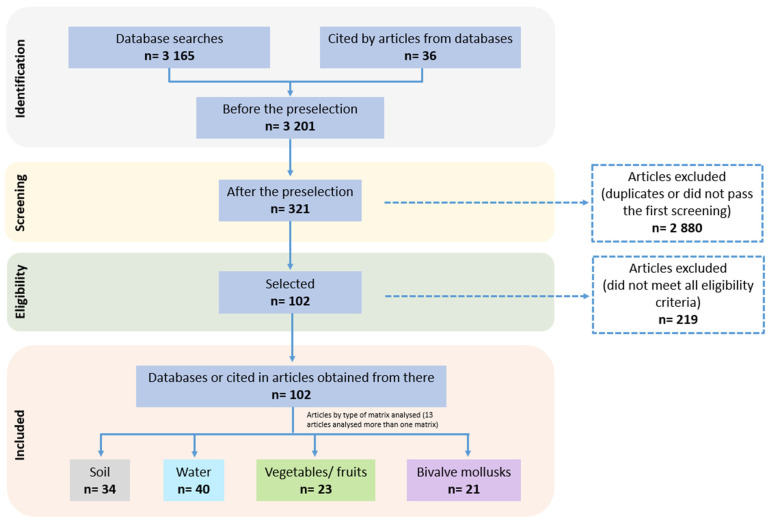
Four-step flow diagram of the systematic review of the presence of *Toxoplasma gondii* oocysts in soil, water, vegetables, fruit, and bivalve mollusks worldwide until the end of 2020.

A total of 3201 articles were obtained from the search process, and 321 were preselected based on their titles or abstract and removal of duplicates. Finally, 102 articles were included for data extraction (Figure 1; Supplementary Table S1). Among them, 13 articles focused on the analysis of two or more matrices and 34 articles reported data on soil, 40 reported data on water, 23 reported data on fresh produce (vegetables and fruit), and 21 reported data on bivalve mollusks. An attempt to gather more data on *T. gondii* oocyst prevalence was undertaken by collecting gray literature (e.g., unpublished scientific information, including reports from governmental agencies, thesis dissertations, conference proceedings) using an online survey administered to experts in the field. The search yielded seven reports not published in English-language peer-reviewed journals with very limited information on the sampling strategies and methodologies employed [42].

### 3.2. Toxoplasma gondii Oocyst Detection in Environmental Matrices

Different environmental matrices have received increasing attention over the past 50 years. The studies included were conducted on soil (*n* = 34) between 1971 and 2019, water (*n* = 40) between 1992 and 2019, fresh produce (*n* = 23) between 2006 and 2019, and bivalves (*n* = 21) between 2002 and 2018. Soil was first investigated early in the 1970s immediately after the full life cycle of *T. gondii* was described and the environmentally resistant stage, the oocyst, was discovered [43]. Later, in the 1990s, the first reports of the presence of *T. gondii* in water were published. More recently, in the 2000s, studies have been conducted in bivalve mollusks and fresh produce.

The timeline of the studies included here appears to be in accordance with our increased understanding of the importance of other food and waterborne zoonotic protists (particularly *Cryptosporidium* spp., but also *Giardia duodenalis* and *Cyclospora cayetanensis*) and the detection of outbreaks. Indeed, from the 1990s onwards, numerous studies demonstrated the presence of *Cryptosporidium* spp. in public water supplies and recreational and river water sources [44], together with two massive outbreaks of cryptosporidiosis in humans associated with water supplies in Georgia and Milwaukee in the United States [45,46], among others. Moreover, water-related toxoplasmosis outbreaks were documented earlier than fresh produce-related outbreaks [14]. Finally, the first studies conducted on mollusks and fresh produce from 2002 or 2006 onwards coincide with similar investigations carried out in other food and waterborne protists. Late in the 1990s, it was reported that bivalves could act as mechanical vectors of *Cryptosporidium* spp. oocysts due to their survival in estuarine waters for several weeks [47], which led to further studies on different bivalve species. Since 2000, both *Cryptosporidium* spp. and *T. gondii* have been more extensively studied in fresh vegetables and fruit [33]. A recent review stated that 5.9% (2/34) of oocyst-related outbreaks were attributable to fresh produce consumption, with both types of fresh produce, vegetables and fruit, as sources of oocysts in outbreaks occurring since 2009 [14].

**Figure 2:**
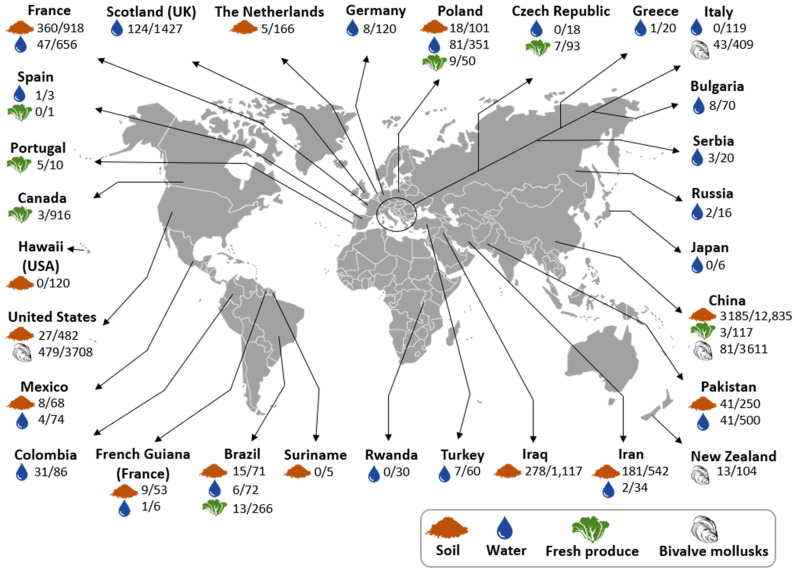
Worldwide detection of *Toxoplasma gondii* oocysts in environmental matrices based on molecular methods (PCR, qPCR, and LAMP) in studies published by the end of 2020. Results are presented as positive samples/total of samples collected. Articles that analyzed pooled samples and did not specify how the number of positive individual samples was estimated were excluded.

*Toxoplasma gondii* was detected in different environmental matrices worldwide using molecular methods (e.g., PCR and loop-mediated isothermal AMPlification, LAMP) or bioassays, which are sensitive and specific methods, as shown in Table 1, Table 2, Table 3 and Table 4 and Figure 2.

The presence of *T. gondii* oocysts in soil was detected in 28 out of 34 studies in the following countries: Brazil (*n* = 5), China (*n* = 7), Costa Rica (*n* = 1), France (*n* = 3), French Guiana (*n* = 1), Iran (*n* = 3), Iraq (*n* = 1), Mexico (*n* = 1), Panama (*n* = 1), Pakistan (*n* = 1), Poland (*n* = 1), the Netherlands (*n* = 1), and the United States (*n* = 2) with overall detection rates ranging from 1.0% (7/700) [48] to 100% (5/5) [49], both from China (Table 1 and Figure 2; Supplementary Table S2).

Water was the environmental matrix most extensively studied worldwide with 25 out of 40 articles reporting *T. gondii*-positive samples in Brazil (*n* = 6), Colombia (*n* = 2), Egypt (*n* = 1), France (*n* = 2), French Guiana (France) (*n* = 1), Germany (*n* = 1), Greece and Bulgaria (*n* = 1), Iran (*n* = 1), Mexico (*n* = 1), Pakistan (*n* = 1), Poland (*n* = 3), Russia and Bulgaria (*n* = 1), Scotland (*n* = 1), Serbia (*n* = 1), Spain (*n* = 1), and Turkey (*n* = 1). Overall, detection rates ranged from 5% (1/20) in Greece [50] to 100% (8/8) in Brazil [51], and most studies reported a detection rate below 20% (Table 2 and Figure 2; Supplementary Table S3).

Altogether, twenty-three studies were conducted on fresh produce matrices that were classified as leafy greens, non-leafy vegetables (including root crops), herbs, and fruit. Positive samples were reported in all fresh produce matrices in 13 articles from Brazil (*n* = 2), Canada (*n* = 1), China (*n* = 1), Colombia (*n* = 2), the Czech Republic (*n* = 1), Egypt (*n* = 1), Italy (*n* = 1), Pakistan (*n* = 1), Poland (*n* = 1), Spain and Portugal (*n* = 1), and Switzerland (*n* = 1) (Table 3 and Figure 2). Detection rates in fresh produce ranged from 0.3% (3/1171) in Canada [52] to 50.0% (13/26) in Portugal [37], and in the majority of studies, detection rates were below 10% (Table 3 and Figure 2, Supplementary Table S4).

Finally, the presence of *T. gondii* oocysts in bivalves was reported in 19 out of 22 studies in Brazil (*n* = 4), China (*n* = 2), France (*n* = 1), Italy (*n* = 3), New Zealand (*n* = 1), Tunisia (*n* = 1), Turkey (*n* = 2), and the United States (*n* = 5), with detection rates that varied from 0.1% (1/1109) [21] to 46.3% (19/41) [53], both in the United States, and from 1.3% (2/160) to 31.0% (19/60) in pooled samples from Brazil [54] and the United States [55], respectively. In most studies, the detection rates were below 7% (Table 4 and Figure 2, Supplementary Table S5).

**Table 1:** Worldwide detection of *Toxoplasma gondii* oocysts in soil based on molecular and bioassay methods in articles published up to the end of 2020.

Sampling Strategy						Methods Used		Results	Sources
Sampling Location (Country)	Sample Origin	No. of Samples Collected	Sample Amount Collected/Sample Size Analyzed (Depth)	Presence of Cats	Link with Human Toxoplasmosis ^a^	Oocyst Recovery Method ^†^	Detection Methods (Molecular Target)	Positive Samples (%)	
Brazil	Dairy farm	5	500 g/500 g (no data)	Yes	Yes	Wash, filtration, centrifugation, flotation, centrifugation, wash, and centrifugation	Mouse bioassay: Sabin Feldman dye test and brain smear confirmed by bioassay in cats	1 (20.0) ^b^	[56]
Brazil	Paddocks from ostrich farms	40	250 g/25 g (5–10 cm)	No data *	No	Wash, filtration, centrifugation, flotation, centrifugation, wash, and centrifugation	PCR, qPCR (529 RE and 18S rRNA)	13 (32.5) ^b^	[57]
Brazil	Elementary public schools	31	1000 g/no data (5 cm)	No data	No	Flotation and centrifugation	Mouse bioassay: squash Mouse bioassay: histopathology Mouse bioassay: immunohistochemistry Mouse bioassay: indirect fluorescent antibody test (IFAT)	7 (22.6) 0 10 (32.3) 8 (25.8)	[58]
Brazil	Sheep farms	10, each inoculated in 5 mice	1 g/1 g (no data)	Yes	No	Wash, flotation, and centrifugation	PCR (529 RE) Mouse bioassay IP/PO- PCR (529 RE) Mouse bioassay IP/PO- IFAT	0 IP: 6 (30.0), PO: 7 (23.3) IP: 14 (70.0), PO: 19 (63.3)	[59]
Brazil	Sludge from a cistern, and soil from greenhouses and vegetable gardens	11	500 mL and 100 g/no data (no data)	Yes	Yes	Centrifugation and flotation	PCR (529 RE)	0	[60]
Brazil	Horticultural properties	10	10 g/10 g (from surface)	Yes	No	Wash and centrifugation	PCR (529 RE)	2 (20) ^b^	[61]
China	Schools, parks, farms, and coastal beaches	2100	20 g/no data (5 cm)	No data	No	Wash, flotation, centrifugation, wash, and centrifugation	PCR, Semi-nPCR, nPCR (529 RE, B1, and ITS-1)	230 (10.9) ^d^	[62]
China	Public parks	252	No data/0.5 g (5 cm)	Yes	No	No data	PCR (B1 and 529 RE) LAMP (MIC3, F3, B3, BIP, FIP, LD, BF)	41 (16.3) ^d^ 58 (23.0)	[63]
China	Pig farms	95	No data/0.5 g (5 cm)	Yes	No	No data	PCR (B1 and 529 RE) LAMP (MIC3, F3, B3, BIP, FIP, LD, BF)	20 (21.1) 36 (37.9)	[64]
China	Urban areas (foci of human habitation, gravel, sand, industrial and commercial land, woodland, grassland)	9420	20 g/4 replicates of 5 g (10 cm)	Yes	No	Wash, flotation, centrifugation, wash, and centrifugation	qPCR (529 RE)	2853 (30.3)	[65]
China	Swine hoggery	5	No data/0.5–5 g (no data)	No data	No	Ultrasonic treatment and sugar flotation	Mouse bioassay: Sabin Feldman dye test and kitten bioassay	5 (100) ^b^	[49]
China	Schools, parks, and grazing area	268	No data/5 g (no data)	No data	No	Wash, filtration, centrifugation, flotation, wash, and centrifugation, presumably	Semi-nested PCR (529 RE)	34 (12.7) ^d^	[66]
China	Chicken farms (free-range and scale farms)	700	10–15 g/10–15 g (from surface)	No data	No	No data	PCR (ITS-1)	7 (1) ^d^	[48]
Costa Rica	Yard and coffee plantation	15	10 g/10 g (from surface or 5–7 cm)	Yes	No	Wash, centrifugation, flotation, centrifugation	Mouse bioassay: Dye test and squash	4 (26.7) ^b^	[67]
France	Areas around a hospital where cats defecate	117	200–300 g/10 g (2 cm)	Yes	No	Wash, filtration, centrifugation, flotation, and centrifugation	qPCR (529 RE)	11 (9.4) ^b^	[68]
France	Village areas, crop field, grassland, forest	243	20 g/4 replicates of 5 g (up to 2 cm)	Yes	No	Wash, flotation, centrifugation, wash, and centrifugation	qPCR (529 RE)	71 (29.2)	[69]
France	Dairy farms	558	20 g/5 g (2 cm)	Yes	No	Wash, centrifugation, flotation, wash, and centrifugation	qPCR (529 RE)	278 (49.8)	[12]
French Guiana (France)	Areas around houses and random sites	53	No data/20 g (no data)	Yes	Yes	Wash and centrifugation	PCR (529 RE)	9 (17.0) ^b,d^	[70]
Hawaii (USA)	University campus and a natural area reserve	120	No data/20 g (10 cm)	Yes	No	Wash, centrifugation, flotation, centrifugation, wash, and centrifugation	PCR (GRA6)	0	[71]
Iran	Urban and rural areas	192	300–500 g/7 g (no data)	Yes	No	Wash, centrifugation, flotation, centrifugation, wash, and centrifugation	nPCR (529 RE)	150 (78.1)	[72]
Iran	Sand pits, playgrounds, public parks, and areas around rubbish dumps	200	400 g/40 g (2–5 cm)	Yes	No	Wash, filtration, centrifugation, flotation, and centrifugation	PCR (GRA6)	18 (9) ^d^	[73]
Iran	Rubbish dumps, children’s playground, parks and public places	150	300 g/no data (3 cm)	No data	No	Wash, centrifugation, flotation, and centrifugation	PCR (B1)	13 (8.7) ^d^	[74]
Iraq	Private gardens, schools, agricultural lands, territory of waste dumps, abandoned lands where children sometimes play, playgrounds, and parks	1117	300 g/40 g (2–5 cm)	Yes	No	No data	nPCR (B1)	278 (24.9) ^b^	[75]
Mexico	Playground boxes	68	10 g/10 g (<2 cm, 2–10 cm or until reaching rock bottom)	Yes	No	Wash, centrifugation, flotation, wash, and centrifugation	nPCR (SAG1)	8 (11.8)	[76]
Panama	Outdoor children’s play areas	924	30 g/30 g (no data)	Yes	Yes	Wash, centrifugation, flotation, and centrifugation	Mouse bioassay: direct agglutination test	10 (1.1)	[77]
Pakistan	Homes, gardens, public enclosures, and backyards from urban and rural areas	250 ^c^	300 g/no data (2–5 cm)	Yes	No	No data	PCR (B1, 529 RE)	B1 = 41 (16.4) ^b^ 529 RE = 41 (16.4)^b^	[78]
Poland	Sand pits, rubbish dumps and sand heaps	101	300 g/40 g (2–5 cm)	Yes	No	Wash, centrifugation, flotation with centrifugation, attachment to a glass slide and wash of the glass slide	PCR (B1 and 200–300 REP)	18 (17.8) ^d^	[79]
Suriname	Different areas from a village	5	200 g/50 g (no data)	Yes	Yes	Flotation (no more information is given)	qPCR (B1)	0	[80]
The Netherlands	Residential gardens and a limited number of playgrounds	166 ^e^	100 g/25 g (5 cm)	No data	No	Magnetic capture	qPCR (529 RE)	5 (3.0)	[81]
The United States	Cities, state parks, public playgrounds, and community gardens	482 ^f^	20–50 g/replicates of 5 g (2–5 cm)	Yes	No	Wash, flotation, centrifugation, wash, and centrifugation	nPCR (ITS1)	27 (5.6) ^d^	[82]
The United States	Pig farms	79	250 g/250 g (no data)	Yes	No	Wash, filtration, centrifugation, flotation, wash, and centrifugation	Mouse bioassay- squash and serology	1 (1.3)	[83]

* Cats were observed near the feed tanks, but no information is provided about their presence in the paddocks, and it is not clear how far the feed tanks were from the sampling area. ^†^ Oocyst recovery method specified step by step. ^a^ Investigations linked with human toxoplasmosis: outbreaks, endemic or sporadic cases (IgG and/or IgM tested and/or clinical signs/symptoms documented). ^b^ Detection rate not given, calculated based on the data provided. ^c^ Stated that 500 soil samples were collected, but results corresponded to 250 samples. ^d^ Positive samples were sequenced and/or genotyped. ^e^ A total of 148 out of 166 samples collected yielded interpretable results by qPCR, but the results were based on the 166 samples collected. ^f^ According to Table 3 of the original manuscript, 501 samples were collected, but 482 samples were considered in the text. Mouse bioassay IP: inoculated intraperitoneally, mouse bioassay PO: peroral. IFAT: indirect immunofluorescence test. LAMP: loop-mediated isothermal amplification. Articles with results only or also based on microscopy assay or other methods that were not specified: [49,57,60,80,84,85,86].

**Table 2:** Worldwide detection of *Toxoplasma gondii* oocysts in water based on molecular and bioassay methods in articles published up to the end of 2020.

Sampling Strategy							Methods Used		Results	Reference
Sampling Location (Country)	Sample Details	No. of Samples Collected	Sample Volume Collected (Sample Volume Analyzed) (Liters—L)	Water Treatment	Presence of Cats	Link with Human Toxoplasmosis ^a^	Oocyst Recovery Method ^†^	Detection Methods (Molecular Target)	Positive Samples (%)	
Brazil	Water from wells	1750 L filtered through 17 membranes and inoculated into 8 chickens	50 per well	No data	No data	Yes (endemic toxoplasmosis area)	Filtration	Chicken bioassay: MATMolecular (no data)	3 (37.5) ^b^ 0	[87]
Brazil	Irrigation and municipal water	3	10	No data	Yes	Yes	Filtration, wash, and centrifugation	PCR (529 RE)	1 (33.3) ^b^	[88]
Brazil	Water from cisterns	3	10–20	No data	Yes	Yes	Filtration, wash, and centrifugation	PCR (529 RE)	0	[60]
Brazil	Irrigation water	10	0.01	No data	Yes	No	Filtration, wash, and centrifugation	PCR (529 RE)	2 (20.0)	[61]
Brazil	Drinking water	4650 L filtered through 56 membranes	No data	Untreated	Yes	Yes	Filtration and centrifugation	PCR (B1) Mouse, chicken, pig and cat bioassays	Positive by at least 1 assay ^c^	[89]
Brazil	Surface water used to produce drinking water	39	20	No data	No data	No	Filtration, wash, and centrifugation	qPCR (B1)	3 (7.7)	[90]
Brazil	Drinking water	8	Given ad libitum to the piglets	Treated (process not specified)	No data	Yes	Directly	Piglet bioassay: IFAT Piglet bioassay: tissue PCR (529 RE) Piglet bioassay: tissue mouse bioassay and PCR (529 RE)	8 (100) 5 (62.5) 5 (62.5) ^b^	[51]
Brazil	Farm water	No data (0.003)	No data	No data	Yes	No	Flotation and centrifugation	PCR (529 RE) Mouse bioassay	No data	[59]
Canada	Untreated water that supplied municipal drinking water treatment plants	11	Mean of 1051	Untreated	No data	Yes	Filtration, wash, centrifugation, flotation, wash, and centrifugation	Mouse bioassay: microscopy from tissue and MAT	0	[91]
Colombia	Water	40	0.2 or 4	Boiled and others not specified	No data	Yes	Sedimentation by centrifugation with formalin-ether	nPCR (B1)	4 (10.0) ^b,c^	[92]
Colombia	Surface water before and during treatment, in the treatment plant network and from homes	46	10	Untreated and treated: coagulation, flocculation, sedimentation, filtration, and chlorination	No data	No	Sedimentation by centrifugation with formalin-ether	nPCR (B1)	27 (58.6) ^c^	[93]
Czech Republic	Irrigation and vegetables washing water	18	10	No data	Not data	No	Filtration, wash, and centrifugation	qPCR (B1 and 529 RE)	0	[94]
Egypt	Irrigation water	54	No data	No data	No data	No	Filtration and centrifugation	Mouse bioassay- smears and MAT	9 (16.7)	[95]
France	Wastewa-ter	35	20	Treated and untreated (process not specified)	No data	No	Filtration, wash, centrifugation, immunomagnetic separation of *Cryptosporidium* spp. and *G. duodenalis*, centrifugation, and flotation	PCR (529 RE)	0	[96]
France	Untreated surface, ground, and public drinking water	139	100 (7–100)	No data	No data	No	Filtration, wash, centrifugation, immunomagnetic separation of *Cryptosporidium* spp. and *G. duodenalis*, flotation, centrifugation, wash, and centrifugation	qPCR (B1) Mouse bioassay- agglutination test and smear	10 (8.0) ^d^ 0	[97]
France	Untreated surface, ground, and public drinking water	482	5–100	No data	No data	No	Filtration, wash, centrifugation, immunomagnetic separation of *Cryptosporidium* spp. and *G. duodenalis*, centrifugation, flotation, and centrifugation	PCR (B1 and 529 RE)	37 (7.7) ^e^	[98]
French Guiana (France)	Water from cisterns, little streams, and brooks	6	10	No data	Yes	Yes	Filtration and presumably wash, centrifugation, flotation, centrifugation, wash, and centrifugation	PCR (529 RE)	1 (16.7) ^b,c^	[70]
Germany	Wastewa-ter	25	1	Untreated and treated: mechanical and biological treatments	No data	No	Filtration (sieve and cellulose filters), wash, and centrifugation	PCR (B1)	0	[99]
Germany	Variable: drinking water and others not specified	95	5–2500	Treated and untreated (process not specified)	No data	No	Flocculation for WWTPs, filtration for drinking, groundwater and surface water, then centrifugation and flotation for samples	LAMP (B1)	8 (8.4)	[100]
Greece Bulgaria Japan	River, reservoir, well, spring, tap, sewage, and recreational water	20 34 6	10	No data	No data	No	Flocculation, centrifugation, discontinuous sucrose gradients, wash, and centrifugation	nPCR (18S rRNA)	1 (5) ^b^ 3 (8.8) ^b^ 0	[50]
Iran	Natural water	34	5	No data	No data	No	Filtration, wash, centrifugation, and flotation	LAMP (B1)	2 (5.8)	[101]
Italy	Wastewa-ter	119	10–20	Sand, membrane-bioreactor, plug-flow reactor, and membrane ultrafiltration	No data	No	Filtration, wash, centrifugation, and flotation	qPCR (B1-multiplex)	0	[102]
Mexico	Public drinking water	74	5	Chlorination	No data	No	Filtration, wash, centrifugation, flotation, centrifugation, wash, and centrifugation	nPCR (SAG1)	4 (5.4)	[103]
Pakistan	Drinking, recreational, and irrigation water	500	No data	No data	No data	No	Flocculation or filtration	PCR (B1 and 529 RE)	41 (8.2) ^b^	[78]
Poland	Drinking water	114	5	No data	Yes	Yes	Filtration, wash, centrifugation, flotation with centrifugation, wash, and centrifugation	PCR (no data)	31 (27.2)	[104]
Poland	Drinking and natural water	201	5	No data	Yes	Yes	Filtration, wash, centrifugation, flotation with centrifugation, wash, and centrifugation	PCR (B1) Mouse bioassay of 14 PCR positive samples-tissue PCR or agglutination test	43 (21.4) ^b,c^ Tissue PCR: 9 (64.3), agglutination test: 3 (21.4) ^b^	[105]
Poland	Bathing and drinking water	36	50	No data	No data	No	Filtration, wash, and centrifugation	nPCR (B1)	7 (19.4) ^c^	[106]
Russia Bulgaria	Natural water	16 36	No data	No data	No data	No	Flocculation, wash, and discontinuous sucrose gradient	nPCR (18S rRNA) LAMP (B1)	2 (12.5) ^f^ 5 (13.9) ^f^ 9 (56.3) ^f^ 16 (44.4) ^f^	[107]
Rwanda	Irrigation and post-harvest washing water	30	1	Untreated those from rivers, lagoons, marshlands, and lakes	No data	No	No data	PCR (529 RE)	0	[108]
Scotland	Public water supply	1427	Up to 1000	No data	No data	No	Filtration, centrifugation, immunomagnetic separation of *Cryptosporidium* spp. and centrifugation	qPCR (529 RE)	124 (8.8) ^c,g^	[109]
Serbia	Surface water from rivers	20	10	No data	No data	No	Filtration, wash, and centrifugation	PCR (529 RE)	3 (15.0) ^c^	[110]
Spain presumably	Irrigation water	3	1.5	No data	No data	No	Centrifugation	qPCR (18S rRNA)	1 (33.3) ^b,c^	[111]
Turkey	Natural water	60	10	No data	No data	No	Flocculation, centrifugation, wash, and discontinuous sucrose gradient	nPCR (18S rRNA) LAMP (B1)	7 (11.7) ^c,h^ 15 (25.0)	[112]
The United States	Presumably drinking water for animals	No data	0.05	No data	Yes	No	Centrifugation	Mouse bioassay-agglutination test and examination	No data	[83]

^†^ Oocyst recovery method specified step by step. ^a^ Investigations linked with human toxoplasmosis: outbreaks, endemic, or sporadic cases (IgG and/or IgM tested and/or clinical signs/symptoms documented). ^b^ Detection rate not given, calculated based on the data provided. ^c^ Positive samples were sequenced and/or genotyped. ^d^ A total of 125 out of 139 samples collected yielded interpretable results. The detection rate was based on the interpretable results. ^e^ A total of 480 out of 482 samples collected yielded interpretable results. The detection rate was based on the interpretable results. ^f^ Reported one detection rate for both countries: nPCR = 7/52 (13.5%), LAMP = 25/52 (48.0%). Detection rates for each country based on the data provided. ^g^ A total of 1411 out of 1427 samples collected yielded interpretable results. The detection rate was based on the interpretable results. ^h^ Six samples were positive by nPCR (mentioned in the abstract); however, detection rates were recalculated according to the results included in the text and tables (7 positive samples). LAMP: loop-mediated isothermal amplification. Articles with results only or also based on microscopy assay or other methods that were not specified: [78,86,96,99,104,105,107,113,114,115,116,117].

**Table 3:** Worldwide detection of *Toxoplasma gondii* oocysts in fresh produce (vegetables and fruit) based on molecular and bioassay methods in articles published up to the end of 2020.

Sampling Strategy								Methods Used		Results	Reference
Sampling Location (Country)	Matrix	Production Type (Organic and/or Conventional)	Product Presentation (Bulk, Packaged or Ready to Eat—RTE)	No. of Samples Collected	Sample Mass Collected (Sample Mass Analyzed)	Presence of Cats	Linked with Human Toxoplasmosis ^a^	Oocyst Recovery Method ^†^	Detection Methods (Molecular Target)	Positive Samples (%)	
Brazil	Lettuce	No data	No data	4	No data	Yes	Yes	Wash, scraping, and centrifugation	PCR (529 RE)	0	[88]
Brazil	Crisp lettuce, regular lettuce, chicory, rocket, and parsley	Organic and conventional	No data	220 ^c^	50 g	No data	No	Wash, filtration, and centrifugation	PCR (B1 and 529 RE)	9 (3.8)	[118]
Brazil	Vegetable clumps (no more details given)	No data	No data	11	50 g	Yes	Yes	Wash, filtration, and centrifugation	PCR (529 RE)	0	[60]
Brazil	Crisp lettuce, arugula, chicory, chives, purple lettuce, spinach, and chard	Organic	No data	42	50 g	Yes	No	Wash, filtration, and centrifugation	PCR (529 RE)	4 (9.5) ^e^	[61]
Canada	Variable ^‡^	Organic and conventional	Bulk and packaged	1171	35 ± 0.5 g	No data	No	Wash, centrifugation, and flotation	qPCR (18S rDNA)	3 (0.3) ^b^	[52]
China	Lettuce, pak choi, Chinese cabbage, rape, asparagus, *Chrysanthemum coronarium*, endive, Chinese chives, cabbage, red cabbage, and spinach	No data	No data	279	No data	No data	No	Wash, flocculation, and centrifugation	qPCR (B1)	10 (3.6) ^b^	[19]
Colombia	Lettuce, cabbage, cucumber, carrot, and tomato	No data	No data	30	200 g	No data	Yes	Wash, sedimentation/centrifugation with formalin ether	nPCR (B1)	1 (3.3) ^b,e^	[92]
Colombia	Strawberries	No data	Bulk and packaged	120	250 g (3 replicates of 30 g)	No data	No	Wash and centrifugation	qPCR (529 RE-multiplex)	6 (5.0) ^b^	[119]
Czech Republic	Carrot, cucumber, lettuce (butterhead lettuce, iceberg lettuce, little gem, and lollo lettuce)	No data	Bulk and packaged (just for lettuce)	292	100 g	No data	No	Wash and centrifugation	qPCR (B1 and 529 RE)	28 (9.6) ^b^	[94]
Egypt	Lettuce, carrot, and cucumber	No data	No data	54	150 g	No data	No	Wash, filtration, and centrifugation	Mouse bioassay: smears + MAT	7 (13.0)	[95]
Italy	Mix salad: curly and escarole lettuce, red radish, rocket salad, and carrots	No data	RTE	648 (72 pools)	100 g	No data	No	Wash and centrifugation	qPCR (B1)	5 (0.8) ^b^	[120]
Pakistan	Apple, banana, guava, cabbage, brinjal, and tomato	No data	No data	250	No data	No data	No	No data	PCR (B1 and 529 RE)	12 (4.8) ^e^	[78]
Poland	Strawberries, radish, carrot, and lettuce	No data	No data	216	1–20 units, 500–1000 g	Yes (in farms-home gardens)	No	Wash, flocculation, and centrifugation	qPCR (B1)	21 (9.7) ^b^	[38]
Spain Portugal	Lettuce, carrot, parsley, watercress, coriander, mix salad, arugula, strawberries, raspberries, and blueberries	Organic and conventional	Bulk, packaged, and RTE	9 26	64–3600 g	No data	No	Wash, centrifugation, immunomagnetic separation of *Cryptosporidium* spp. and *G. duodenalis*	PCR (529 RE)	2 (22.2) ^b,d^ 13 (50.0) ^b,d^	[37]
Switzerland	Lettuce (different types, but not specified)	No data	No data	100	900–1800 g (pools of 9 lettuce)	No data	No	Wash, filtration, and centrifugation	PCR (B1)	6 (6.0) ^b,e^	[121]

^†^ Oocyst recovery method specified step by step. **^‡^** Types of fresh produce analyzed: arugula/baby arugula, kale, spinach/baby spinach, romaine, chard, leaf lettuce (green and red), spring mix, leafy green mixes (mix of 2 or more leafy green types), any dandelion, collards, rapini, escarole and marche. ^a^ Investigations linked with human toxoplasmosis: outbreaks, endemic, or sporadic cases (IgG and/or IgM tested and/or clinical signs/symptoms documented). ^b^ Positive samples were sequenced and/or genotyped. ^c^ According to the abstract, a total of 238 samples were collected, but the sum of each type of vegetable collected corresponded to 220 samples. ^d^ Fourteen positive samples were reported in the text, but there were 15 positive samples in the tables, and detection rates by country were not given. ^e^ Detection rate not given, calculated based on the data reported. Articles with results only or also based on microscopy assay or other methods that were not specified: [120,122,123,124,125,126,127,128,129].

**Table 4:** Worldwide detection of *Toxoplasma gondii* oocysts in bivalve mollusks based on molecular and bioassay methods in articles published up to the end of 2020.

Sampling Strategy					Methods Used		Results	Reference
Sampling Location (Country)	Sample Type (Scientific Names)	Samples Collected	Sample Units per Pool or Sample Mass (Length)	Type of Tissue or Material Analyzed	Oocyst Recovery Method ^†^	Detection Methods (Molecular Target if Apply)	Positive Samples (%)	
Brazil	Oysters (*Crassostrea rhizophorae*), mussels (*Mytella guyanensis*)	80 pools	5–15 units/pool	Whole oyster or mussel	Wash, filtration, centrifugation, wash, and centrifugation	nPCR (B1) Mouse bioassay- smear + IFAT	2 (2.5) ^a,b^ 0	[130]
Brazil	Oysters (*Crassostrea rhizophorae*)	208 pools of each tissue	3 units/pool (no data)	Gills and digestive glands	Not performed *	PCR (529 RE) nPCR (SAG1)	0 17 (8.1) ^b^	[131]
Brazil	Oysters (*Crassostrea* spp.)	120 pools	10 units/pool (no data)	Gills, gastrointestinal tract, and intervalvular liquid	Not performed *	nPCR (B1)	7 (5.8) ^b^	[132]
Brazil	Oysters (*Crassostrea* spp.)	80 pools of each tissue	5 units/pool (no data)	Gills and digestive glands (visceral mass)	Not performed*	nPCR (SAG1)	2 (2.5) ^b^	[54]
China	Oysters (not specified)	998	1 unit (no data)	Hemolymph, digestive glands and gills	Centrifugation	Semi nPCR (B1)	26 (2.6) ^b^	[133]
China	Mussels (*Mytilus edulis*)	2215	1 unit (no data)	Gills, digestive glands and hemolymph	Not performed *	Semi nPCR (B1)	55 (2.5) ^b^	[134]
China	Oysters (*Concha ostreae*)	398	1 g/sample (no data)	Digestive tract tissues	Not performed *	PCR (ITS1)	0	[135]
France	Mussels (*Dreissena polymorpha*)	96 pools	9 units/pool (18–25 mm)	Whole mussel	Enzyme digestion, centrifugation	qPCR (529 RE)	3 (3.1)	[136]
Italy	Mussels (*Mytilus galloprovincialis*)	409	25 mg (>5 cm)	Digestive gland	Not performed *	qPCR (B1)	43 (10.5) ^b^	[137]
Italy	Mussel (*Mytilus galloprovincialis, Mytilus edulis*)	135 pools	10 g (no data	Intestinal tissues	Wash, filtration, centrifugation, wash, and centrifugation	End-point PCRs (B1 and 529 RE)	10 (7.4) ^b^	[138]
Italy	Oysters (*Crassostrea gigas*)*,* mussels *(Mytilus galloprovincialis*), clams *(Tapes philippinarum, Tapes decussatus*)	62 pools of each tissue	11–30 units/pool (no data)	Digestive glands, gills and hemolymph	For hemolymph: flotation, centrifugation, wash, and centrifugation. Not specified for digestive glands and gills	nPCR and FLAG- qPCR (B1)	2 (3.2)	[139]
New Zealand	Mussels (*Perna canaliculus*)	104	1 unit (no data)	Hemolymph	Centrifugation	nPCR (dhps)	13 (12.5) ^b^	[23]
Tunisia	Clams (*Ruditapes decussatus*), oysters (*Pinctada radiata*), mussels (*Mytilus galloprovincialis, Perna perna*)	87 pools	9–18 units/pool (no data)	No data	Wash, filtration, centrifugation, wash, and centrifugation	qPCR (B1)	4 (4.6) ^a,b^	[140]
Turkey	Mussels (*Mytilus galloprovincialis*)	53 pools	15 units/pool (5–8 cm)	Gills and digestive system	Filtration and centrifugation	qPCR (B1) + HRM	5 (9.4) ^b^	[141]
Turkey Italy	Mussels (*Mytilus galloprovincialis)*	53 pools 60 pools	15 units/pool (no data) 500 g (no data)	Gills and digestive system Hemolymph, gills and digestive glands	Flotation or filtration and centrifugation	qPCR + HRM (B1)	7 (13.2) 0	[102]
The United States	Oysters *(Crassostrea virginica*)	1440	50–100 mg wet weight of total tissue (no data)	Mantle, gills and rectum	Not performed *	qPCR (ITS1)	446 ^a^ (31.0)	[55]
The United States ^‡^	Mussels *(M. californianus*)*,* gaper clams (*Tresus nuttallii*)*,* pismo clams (*Tivela stultorum*)	1109	50 mg of digestive tissue or 50–100 μL of pelleted hemolymph (no data)	Hemocytes and digestive gland	Not performed *	qPCR (18S rRNA)	1 (0.1) ^a,b^	[21]
The United States ^‡^	Mussels (*Mytilus californianus*)	959	1 unit (no data)	Hemolymph	Centrifugation	nPCR (ITS1 and B1)	13 (1.4) ^b^	[22]
The United States	Mussels (*Mytilus* spp.)	41	1 unit (no data)	Hemolymph, gills and digestive glands	Filtration and centrifugation	qPCR and end- point PCR (529 RE)	19 (46.3) ^a,b^	[53]
The United States	Clams (*Mya arenaria*), mussels (*Geukensia demissa, Mytilus edulis*), oysters (*Crassostrea virginica*)	159	1 unit (no data)	Digestive gland, mantle, gills, foot, and siphon	Not performed *	PCR (GRA6)	0	[142]
The United States	Mussel (*Mytilus californianus*)	Analyzed pools, but the exact number was not Specified (total of units = 959)	30 units/pool (≥3 cm)	Hemolymph	Not performed *	PCR (ITS1, 529 bp and B1)	13 (1.5) ^b,c^	[117]

^†^ Oocyst recovery method specified step by step. **^‡^** Presence of cats in the sampling area reported by previous studies. * Samples were analyzed without a preceding oocyst recovery/concentration process. ^a^ Positive samples or detection rates not specified, calculated based on the data provided. ^b^ Positive samples were sequenced and/or genotyped. ^c^ Detection rate based on the total of individual samples collected, not based on analyzed pools. HRM: high-resolution melt curve. FLAG: fluorescent amplicon generation. IFAT: indirect immunofluorescence test. None of the articles were linked to human toxoplasmosis.

Most studies were focused on a few countries, so data cannot be extrapolated to other areas. The fact that most of the studies included in this systematic review were performed in North and South America could be linked to the frequency of oocyst-associated toxoplasmosis outbreaks, which were documented as early as 1966 in these regions [143]. Brazil is the country most represented in the studies, which is likely because it is a hotspot for outbreaks and the presence of a wide variety of strains that appear more virulent [7].

It is noticeable that very few studies addressed *T. gondii* infection using a multisectoral and transdisciplinary approach, according to the One Health concept. Indeed, only 13 of the selected articles studied the association between oocyst detection in environmental matrices with human *T. gondii* infection and toxoplasmosis (outbreaks, endemic, or sporadic cases), most of them from North and South America. Five of these studies focused on soil [56,60,70,77,80], with three of them reporting positive samples; 10 were in water [51,60,70,87,88,89,91,92,104,105], with eight reporting positive samples; and three were on fresh produce [60,88,92], with one reporting positive samples and the other reporting negative samples, but suggesting that the occurrence of toxoplasmosis was connected with vegetable consumption in a restaurant [60] (Table 1, Table 2, Table 3 and Table 4).

### 3.3. Sampling Strategies

The studies selected were not comparable due to the large differences between them. When analyzing pooled detection rates by matrix type, a high degree of heterogeneity was observed (I^2^ = 98.9%, *p* < 0.001) due to the different sampling and methodological approaches adopted among the 64 studies included here (Table 5). Fresh produce stood out as the least heterogeneous matrix (I^2^ = 78.2%, *p* < 0.001). Nevertheless, this might be a consequence of the small number of studies selected (*n* = 8) because most of the available surveys analyzed pooled samples and were excluded. A larger number of studies (*n* = 28) were considered for water. However, high heterogenicity was obtained (I^2^ = 85.4%, *p* < 0.001) even though sampling strategies were adopted from standardized protocols for other waterborne parasites such as *Cryptosporidium* spp. and *G. duodenalis* [144]. As expected, similar results were found when analyzing heterogeneity by Cochran’s chi-squared (Q = 6679.21 (d.f. = 74), *P* < 0.001). In addition, the first approach to estimate the sampling bias showed a significant influence (Egger’s test = 4.41, *p* < 0.001), which provides additional statistical evidence of heterogeneous sampling strategies and methodologies [41,145] (Table 1, Table 2, Table 3 and Table 4; Supplementary Figures S1–S4). Such bias was also evident in the funnel plots constructed for each of the matrices (Supplementary Figure S5). Nevertheless, we did not exclude any of the studies aiming to show a detailed overview of the investigations carried out up to date. Thus, harmonized procedures should be implemented in future studies.

#### 3.3.1. Soil

Soil samples were grouped into different categories according to their origin, which was mainly based on their proximity to urban areas and the presence of domestic and wild felids: public parks and playgrounds, schools, gardens, backyards, and houses (including vegetable gardens/orchards), livestock farms, crop fields and grasslands, and forests (Table 1, Supplementary Table S2). In general, the detection rates in soil near urban areas were between 1.1% (10/924) in playgrounds [77] and 94.1% (16/17) in vegetable gardens [72]. On livestock farms, detection rates ranged from 1.0% (7/700) [48] to 100% (5/5) [49], in crop fields and grasslands from 20.0% (2/10) [61] to 32.4% (274/845) [65], and in forests from 32.1% (26/81) [69] to 85.7% (6/7) [72] (Supplementary Table S2).

The higher detection rates reported in livestock farms, vegetable gardens, and forests may be explained by the presence of felines, since 20 out of 23 articles that documented the presence of cats near the sampling area also reported positive samples. There is evidence that *T. gondii* oocyst contamination is more common at known cat defecation sites than at other sites [68], and in farms with higher cat densities [63]. In a study in eastern France, soil contamination with oocysts decreased as the distance from core areas of cat home ranges increased [69]. In studies reporting the presence of cats, the detection rates ranged from 1.1% (10/924) [77] to 78.1% (150/192) [72], whereas they ranged from 1.0% (7/700) [48] to 100% (5/5) [49] in studies where no information was provided. For further studies that aim to determine the risk that the presence of cats poses to *T. gondii* environmental contamination, quantitative data on cat colonies would help to better interpret the results obtained.

The prevalence of oocyst-shedding cats may vary with seasonal reproductive patterns, and the likelihood of exposure to *T. gondii* may be influenced by climatic conditions [62]. There is evidence that season and extreme weather events are variables that influence *T. gondii* contamination. *T. gondii* oocysts remain viable for a long period of time in moist soil conditions and mild temperatures. For example, significant levels of rainfall may lead to humidity, precipitation, and excess runoff, and thus, exposure to *T. gondii* oocyst is increased [12,21]. In contrast, drier conditions and hot temperatures reduce the persistence (and viability) of *T. gondii* oocysts in the soil [82,146]. A handful of studies have investigated the effects of climate conditions and season on soil contamination with *T. gondii* oocysts. Soil, temperature, and humidity were found to be associated with oocyst contamination in Harbin, China [65]. In another study from China, soil contamination was more common in a sub-tropical climate [62]. In three studies, oocyst positive soil samples were found more frequently in autumn [48,62,82]. In contrast, a gradual decrease in soil detection rates from spring to winter was reported in Wuhan, China [63], and levels of soil contamination were higher in the summer season than in the spring in Mazandaran Province, Iran [72]. Local variations in climate may explain the seasonal differences observed, and this highlights the importance of recording climatic conditions when undertaking environmental sampling.

Other sampling variables to be considered are the number of samples collected that ranged from 5 [56] to 9420 [65], the mass of soil sampled that varied from 1 g [59] to 1000 g [58] and the sampling depth that ranged between 2 and 10 cm (Table 1). However, this information was not provided in some articles, and thus, comparison between articles was not possible.

Currently, there is a lack of knowledge on the nature or extent of any effect of soil type on *T. gondii* oocyst survival. The biological, chemical, and physical parameters of soil may vary with soil type and sampling season and therefore affect oocysts’ viability, recovery, and detection. An experimental study conducted with *T. gondii* oocysts and different types of artificial and natural soil matrices demonstrated that the efficiency of oocyst recovery is affected by the soil characteristics, with significantly higher efficiency from samples that had the lowest sand content [147], which was probably due to the structural damage caused by mixing before and during the flotation procedure, as documented previously for *Cryptosporidium* spp. [148]. Therefore, all these parameters should be documented to facilitate the development of risk assessment and management strategies aimed at detecting *T. gondii* oocysts, estimating the environmental contamination burden, and reducing public health risks [62].

#### 3.3.2. Water

Regarding investigations on the occurrence of *T. gondii* oocysts in water, specific sampling variables were considered: the water origin (groundwater: wells; surface: rivers, beaches, lakes, pools; wastewater; piped water: from homes or public drinking water), uses (recreational: swimming and/or playing sports; irrigation/washing; potable water) and water treatment (boiling, chlorination, filtration) (Table 2, Supplementary Table S3).

*Toxoplasma gondii* detection rates ranged from 5.4% (4/74) [103] to 37.5% (30/80) [104] in groundwater water, 5.0% (5/100) [98] to 76.9% (10/13) [93] in surface water, 10.0% (1/10) [50] to 42.9% (3/7) [107] in wastewater, and 2.3% (1/44) [97] to 17.9% (5/28) in piped water. Lower *T. gondii* detection rates in drinking water and groundwater compared to surface water have been reported previously [78]. This could be due to the water treatment received or natural filtration through soil, stones, and organic matter, respectively. However, this last hypothesis will depend on the characteristics of the ground, since material of smaller diameter could retain more oocysts, as experimentally proven for *Cryptosporidium* spp. oocysts [149]. It is also possible that inhibitors that might affect molecular tests are more likely present in groundwater or wastewater, leading to an underestimation of the contamination with oocysts. Surface water may be directly in contact with definitive host feces or accumulated rainfall runoff from surfaces, leading to higher oocyst contamination.

The public health importance of different contaminated water sources is determined by their uses. In relation to this, one article reported 9.0% (9/100) recreational water samples to be oocyst positive [78], while the detection rates were between 16.7% (9/54) [95] and 50.0% (1/2) [88] in irrigation/washing water, and 2.3% (1/44) [97] and 100% (8/8) [51] in potable water. The origin of these samples was not specified in all cases; the recreational water corresponded to lakes and pools, the irrigation/washing water was from a river, and in one study, the potable water corresponded to water kept in tanks/towers from houses, in fountains, and from the water and sewage company. One study with 100% of positive potable water samples was linked to a human toxoplasmosis outbreak in Santa Maria, Brazil, which was one of the largest studies worldwide with around 902 confirmed cases [51] and one of the few that used piglet bioassay for parasite detection; thus, oocysts infectivity was confirmed.

Previous studies have stated that untreated surface irrigation water is a relevant source of waterborne pathogens including *T. gondii* [111] and that human *T. gondii* infection seropositivity is significantly more frequent among those consuming unboiled water [104]. Herein, three out of eight articles that analyzed treated water clearly specified the treatment received, all from North and South America, with detection rates of 5.4% (4/74) in chlorinated water [103], 6.0% (2/30) in boiled water [92], and 60.0% (12/20) in water from the distribution system of a treatment plant after a coagulation, flocculation, sedimentation, filtration, and chlorination process [93]. However, oocyst viability was not assessed in these studies, and it is documented that sporulated oocysts lose their infectivity at 60 °C for 1 min [150], while treatments such as chlorination, ultraviolet (UV), and ozone are not effective [151]. One study that analyzed treated water reported 100% (8/8) of positive samples by piglet bioassay, which were linked to a human toxoplasmosis outbreak, but water treatment was not specified [51]. This finding could indicate that not all treatments used are effective or that treated water may become contaminated, which is more likely in countries with inadequate water supply systems. Thus, in addition to avoiding the contamination of stored water (tanks, cisterns, and others), the effectiveness of treatments and the post-treatment handling are both crucial factors to be considered in the prevention and control of water-related toxoplasmosis.

The number of samples collected was extremely variable: from three irrigation, municipal, and/or cistern water samples [60,88,111] to 1427 public water supply samples [109]. In addition, the sample volume ranged from 0.01 L [61] to up to 2500 L [100]. The analysis of large volumes is necessary because of the low oocyst load expected. However, water turbidity due to organic matter can also have an impact, since this can lead to membrane saturation [70] and increase the possibility of the presence of inhibitors affecting the molecular tests.

Altogether, nine articles recorded the presence of cats in the sampling area, and six of them reported positive samples [61,70,88,89,104,105] (Table 2). However, no clear association was established. One article suspected that reservoir contamination was due to a cat from the area that gave birth to kittens that lived on the top of the reservoir, but they could not be caught, and so it was not possible to confirm this hypothesis [89]. Since most of the studies did not specify whether cats were present, it was not possible to determine if reported detection rates were influenced by this variable. Moreover, the presence of cats alone is insufficient to explain the results, since infected cats can shed oocysts that could contaminate areas located far away from the sampling sites through water currents.

Detection rates by sampling season were not documented in most studies. One study in Scotland (UK) reported a higher number of positive samples in autumn compared to summer [109], and other studies in Mexico and Brazil found positive samples only during the rainy season [90,103]. In addition, a study in French Guiana, linked to a human toxoplasmosis outbreak, stated that climate changes, mainly flooding and warming, were the prelude to the event [70]. Therefore, it seems that there is a positive association between wet seasons and the presence of oocysts in water samples; however, further studies are required to confirm this. A higher probability of detecting *Cryptosporidium* spp. and *G. duodenalis* in fresh surface water during and after extreme weather events has been also documented, with mean odds ratios of 2.61 (95% CI = 1.63–4.21) and 2.87 (95% CI = 1.76–4.67), respectively [152]. Accordingly, apart from classical climatologic parameters such as temperature and humidity, extreme weather events might strongly influence oocyst presence, and this should be taken into account in risk assessments.

#### 3.3.3. Fresh Produce

Regarding fresh produce, sampling strategies varied in terms of matrix composition, number and mass of samples, season, origin (growing location: gardens/orchards, open fields, green houses; market: local markets and fairs, supermarkets, or restaurants), production type (organic versus conventional), and product presentation (bulk, prepacked, and RTE) (Table 3, Supplementary Table S4).

Fresh produce is a heterogeneous matrix that was divided into four distinct categories in this study: leafy greens including mixed salads (*n* = 13), non-leafy vegetables including root crops and others (brinjal, asparagus, beet, radish, carrot, chives, chili, tomato; *n* = 9), herbs (basil, dill, chicory, coriander, thyme, parsley; *n* = 3), and fruit (apple, banana, guava, blueberry, raspberry, and strawberry; *n* = 3). Leafy greens included different types of lettuce, chicory, rocket (syn. Arugula), watercress, chard, spinach/baby spinach, and *Brassica* vegetables (cabbage, red cabbage, rape, pak choi). Lettuce was investigated in 12 studies, but a harmonized nomenclature was not found, since the type of lettuce was not specified in seven studies, whilst in others, the authors mentioned romaine, red and green leaf lettuce, regular lettuce, curly lettuce, butterhead lettuce, iceberg (syn. Crisp) lettuce, little gem, lollo lettuce, escarole, or simply “varieties of lettuce”. In addition to this, 13 studies analyzed one type of sample, whereas three studies analyzed a mix of vegetables. The composition of fresh produce might influence the possibility of being contaminated with oocysts, since the production process and growing period differ notably between baby leaves, grown and cut leafy greens, roots, and fruit.

According to the product presentation, three studies analyzed a mixture of leafy greens, and two of them were specifically RTE products, with detection rates of 0.8% (5/648) [120] and 33.3% (2/6) [37]. Apart from these mixed salads, samples of arugula and watercress were also RTE, with 66.7% (2/3) of positive samples only in the last case [37]. The differences in detection rates may be at least partially explained by the number of samples collected and tested. It is clear that current RTE production processes do not guarantee a product free from parasites of fecal origin, as not only *T. gondii* but also *Cryptosporidium* spp., *G. duodenalis*, *Cyclospora cayetanensis*, *Blastocystis hominis*, and *Dientamoeba fragilis* have been detected in RTE products, [120]. By contrast, a recent study performed in Italy did not detect *T. gondii* in 324 locally produced RTE mixed salads [153]. However, *Echinococcus granulosus* was detected in one sample, providing evidence for the risk of being contaminated with parasites of fecal origin [153].

The number of samples analyzed varied from one study to another, from one arugula, spinach, or chard sample [37,61] to 387 baby spinach samples [52], and three pools of cabbages [92] to 100 pools of lettuces [121] for leafy greens, from five chive [61] to 18 asparagus samples [19], and one pool of carrots [37] to 109 pools of cucumbers [94] for roots and other types of fresh produce, from three coriander [37] to 16 *Chrysanthemum coronarium* samples for herbs [19], and from two pools of raspberries or blueberries [37] to 120 pools of strawberries for fruit [119]. Moreover, six [37] to 648 mixed salad samples were analyzed [120]. In general, the sample amount ranged from one to 20 units or 35 to 3600 g. Since no validation data of the detection methods used for pooled samples were reported, pooling may also have influenced the sensitivity of the detection assays.

Seasonal oocyst detection rates were reported in a few studies on fresh produce with inconclusive results. One study reported a higher detection rate in autumn compared to summer in Switzerland [121], while others reported higher detection rates in samples collected in summer [19,120] or in autumn/winter [37], but in most of these studies, there were no significant differences in detection rates. As discussed previously, extreme weather effects should be also recorded.

Altogether, four articles specified the type of production, and *T. gondii* was detected in both organic and conventional fresh produce [37,52,61,118]. One study performed statistical analysis and reported no significant differences between the two types of production [37]. Similarly, no significant differences were reported between conventional and organic samples in a recent study performed in the United States [154]. Moreover, the sampling locations were diverse, and *T. gondii* was detected in samples collected from open fields, community fairs, storage, local markets, farmlands, school restaurants, and supermarkets [19,37,52,61,92,118]. A relevant issue that could favor or hamper oocyst contamination through cat feces may be growing the vegetables in open fields vs. in greenhouses. Unfortunately, although a greenhouse origin was recorded for one study [19], whether the vegetables were grown in open fields or in greenhouses was not specified in other studies.

Data on water sources, irrigation systems, and types of fertilization was limited or not provided in studies included here. Nevertheless, these variables have been identified as relevant risk factors for other foodborne pathogens and may explain some figures reported by the EFSA [155]. A study from the Czech Republic specified that vegetables were irrigated with water from rivers, lakes, or wells and washed with water from the distribution system or wells [94]. Another study from Egypt also stated that the vegetables were irrigated with water from river canals [95]. Water samples were tested in both studies, and *T. gondii* was detected in one of them [95], while oocysts were detected in fresh produce in both cases (Table 3). The presence of *T. gondii* oocysts in soil and surface water used for fresh produce production and processing (including packinghouse operations) suggests that there may be a risk of contamination of these products, as previously reported for *Cryptosporidium* spp. and *G. duodenalis* in the fresh produce industry [156]. Organic fertilizer (compost/sludge/manure) might not pose a major risk for *T. gondii* since cat feces are not usually used as fertilizer. In contrast, it is likely that access of cats to crops and weather events spreading oocysts pose a risk for the presence of *T. gondii* oocysts on fresh produce. Thus, oocyst contamination is more unlikely to occur in greenhouses vs. open fields, where the access of cats can be more easily restricted and fresh produce is protected from weather events. One study stated that vegetables grown close to farms are at higher risk of *T. gondii* contamination, which is probably due to the presence of felines [38]. The presence of cats was a variable recorded in four of the selected studies [38,60,61,88], but only two of them reported positive samples [38,61].

#### 3.3.4. Bivalve Mollusks

Different genera of clams, oysters, and mussels were analyzed worldwide to determine the presence of *T. gondii*. Detection rates varied between 3.6% (1/28) [139] and 6.6% (4/61) [140] in pooled clams (*Ruditapes decussatus*) from Italy and Tunisia, respectively, 1.3% (2/160) [54] and 31.0% (446/1440) [55] in different species of pooled or individual oysters (*Crassostrea* spp.) from Brazil and the United States, 1.4% (13/959) [22] and 46.3% (19/41) [53] in individual samples of different species of mussels (*Mytilus* spp.) from the United States, and 3.1% (3/96) in pooled zebra mussels (*Dreissena polymorpha*) from France [136] and 12.5% (13/104) in individual samples of New Zealand mussels (*Perna canaliculus*) from New Zealand [23] (Table 4, Supplementary Table S5).

It has been reported that filtering activity is multifactorial and affected by bivalve genera, variations in salinity (fluctuates more in coastal marine areas), temperature, level of contamination, and kinetics of parasite diffusion, among other factors [18]. In the studies reviewed here, the same genera of bivalves were collected in different parts of the world, in different locations (coastal, bay or beach, farms and depuration plants, markets/outlets, water treatment plant discharge points), seasons, and at different depths. Moreover, there were other variations in sampling, e.g., pooled samples vs. individual samples, and different tissues selected for oocyst detection. This variation hampers comparisons between studies.

Regarding sampling location, detection rates varied from 1.4% (13/959) in *Mytilus californianus* samples [22] to 31.0% (446/1440) in *Crassostrea virginica* [55] samples from coastal sites in the United States, 1.3% (2/160) [54] to 16.6% (1/6) [139] in pools of *Crassostrea* spp. from farms in Brazil and Italy, respectively, 2.5% (55/2215) to 12.5% (13/104) in individual samples of *Mytilus edulis* [134] and *Perna canaliculus* [23] from markets/outlets in China and New Zealand, respectively, and 3.1% (3/96) in pools of *Dreissena polymorpha* from different discharge points of wastewater treatment plants in France [136]. The latter study was the only one that documented the depth of the sample collection, which was 20–100 cm. The results from the coastal sites may be influenced by several aspects, including seasonality, human settlements due to the presence of domestic cats, and industrial wastewater. Proximity to freshwater runoff has been associated with the presence of pathogens, including *T. gondii*, in bivalves [117].

In relation to seasonality, *T. gondii* oocyst contamination in bivalves was more frequent in spring and autumn compared to other seasons in Italy [138], in summer compared to winter in New Zealand [23], and during the wet season in the United States [22,117]. This could be explained by region-specific weather patterns in each country, so that higher contamination coincides with the rainy season. Extended dry periods may lead to a greater accumulation of oocysts on land that can be mobilized into runoff in subsequent periods of heavy rainfall.

The anatomical regions analyzed were documented in some articles that investigated the presence of *T. gondii* based on molecular assays, but few of them reported the positive samples by tissue type. Most studies analyzed the digestive glands, followed by gills and the hemolymph (Table 4). The digestive glands and hemolymph seem to be appropriate target organs according to the few spiking studies done. *Toxoplasma gondii* was most often detected in digestive glands compared with hemolymph or gills after experimental contamination under laboratory conditions carried out in mussels [157]. In a later experimental infection done in zebra mussels followed by a depuration process, the greatest concentrations of *T. gondii* DNA were observed in hemolymph and mantle tissues [158]. In field studies, there is evidence that oocysts are more frequently detected in the digestive system and/or hemolymph than in the gills [134,141]. However, other studies only found positive gills [54,139] or a higher frequency of positive gills compared to digestive samples [131]. Thus, the three tissues, or at least gills and digestive glands, should be analyzed independently or in pools to optimize parasite detection, since oocyst concentration may vary in these tissues with time post-infection [157]. Indeed, it is recommended to pool the gills and digestive tract, since this strategy optimizes parasite detection in mussels and clams based on the literature published in the presence of *T. gondii,* as well as *Cryptosporidium* spp. and *G. duodenalis*, in marine mollusks [18]. Conversely, other researchers suggest the use of hemolymph instead of all tissues due to the presumed presence of lower levels of inhibitors and less viscous material, which may improve the sensitivity of the technique [22].

Another important sampling variable is the number of samples analyzed, which varied from 41 [53] to 2215 [134] samples, and from 53 [141] to 208 [131] pools composed of three [131] to 30 [139] units per pool. On the other hand, only a handful of articles (*n* = 3) reported the length of the samples collected, which were longer than 5 cm in the case of *Mytilus galloprovincialis*, with similar detection rates in two studies, 10.5% (43/409) of individual samples [137] and 9.4% (5/53) of pools [141], and 18–25 mm in the case of *Dreissena polymorpha*, with 3.1% (3/96) of positive pools [136].

None of the studies determined the relationship between the presence of oocysts in bivalves and human toxoplasmosis cases caused by their consumption. There is no estimate of the number of *T. gondii* outbreaks associated with consumption of shellfish, including bivalves. According to a study performed in the United States, the consumption of raw oysters, clams, and mussels was identified as a risk for recent *T. gondii* infection (OR = 2.22, 95% CLs 1.07–4.61) [159].

Although the viability of the oocysts detected is unknown, and the only attempt to isolate *T. gondii* by mouse bioassay was not successful [130], bivalves cannot be ruled out as a potential source of infection to humans when they are consumed raw or undercooked. Moreover, it was previously suggested that the sampling strategy should focus on edible mollusk species raised under controlled conditions to better estimate the load and infectivity of filtered parasites that may pose a risk for consumers [18].

### 3.4. Toxoplasma gondii Oocyst Detection Methodology

A lack of harmonized methods for detecting *T. gondii* oocysts was observed in all environmental matrices (Table 1, Table 2, Table 3 and Table 4), and it has been extensively discussed for fresh produce [9]. This issue is supported by the high degree of heterogeneity reported in Section 3.3, and it is also reflected in the range of analytical sensitivities reported for the detection of spiked oocysts in environmental samples in a limited number of studies. In soil samples, ranges of 10–1000 oocysts or 5–50 tachyzoites could be detected in 1 to 300 g by PCR, qPCR, and nPCR [12,66,79]. The analytical sensitivity in water was 1–1000 oocysts per L by PCR and qPCR [90,97,98]. In the case of fresh vegetable samples, the sensitivity reported was 10 oocysts per 30 g of sample [119], and the number of spiked oocysts detected in bivalve tissues was in the range of 5–1000 oocysts in hemolymph per mL or sample by nPCR [22,23] and 100 oocysts in gill tissue by real-time PCR [53].

The recovery of *T. gondii* oocysts and parasite detection are two key sequential steps. Higher variability among different matrices was observed regarding oocyst recovery compared with oocyst detection methods. In fresh produce and bivalve mollusks, a first key point for oocyst recovery was the sampling of individual vs. pooled samples. The analysis of pooled samples may facilitate oocyst detection [120,139], but the recovery and detection methods should be standardized in order to determine the maximum number of samples included in the pool to detect the minimum number of oocysts established by spiking assays. Accordingly, spiking experiments are highly recommended to evaluate the oocyst recovery rate as well as PCR analytical sensitivity and specificity in these complex matrices.

Second, the most extensively used recovery methods for soil and fresh produce were a combination of washing and centrifugation steps that may also include filtration or flotation. Large volumes can be a limiting factor, and filtration has been suggested for fresh produce when working with large volumes of wash buffer or samples [9]. Filtration was the preferred method for water and bivalve samples in combination with centrifugation and/or flotation, although the direct analysis of samples, without a previous oocyst recovery/concentration procedure, was also frequent in bivalves (Table 1, Table 2, Table 3 and Table 4). A filtration–centrifugation method is the basis of the official USEPA method 1623 recommended by the U.S. Environmental Protection Agency (EPA) to evaluate waterborne parasites, such as *Cryptosporidium* spp. and *G. duodenalis*, in drinking water. However, this method also includes an immunomagnetic separation (IMS) step with specific commercial antibodies. A few specific polyclonal antibodies directed against *T. gondii* oocyst wall components have been generated that could be used for this purpose [160,161], but unfortunately, there is no commercially available anti-*T. gondii* oocyst antibody. Although several IMS methods have been developed [162,163], improvement of the recovery rate with IMS needs to be demonstrated for environmental matrices [18]. A recent study has achieved a proof-of-principle method for oocyst capture and separation from water using lectin magnetic separation that was later followed by qPCR, and this could be considered in future studies [164].

Recovery efficiency can be also influenced by the formation of foam that can be a challenge in handling fresh produce matrices rich in saponins [9]. It is unclear how different buffers employed for oocyst recovery could work with the different matrices and with the different mixes of vegetables analyzed. Thus, the avoidance of detergents in washing buffers (at least for fresh produce) should be considered. In order to confirm the use of appropriate buffers and efficient separation methods, spiking experiments with oocysts should be done in order to maximize the efficiency of oocyst recovery during the method standardization process [165].

Regarding detection methods, PCR was extensively employed and predominated vs. microscopy and bioassay methods (Table 1, Table 2, Table 3 and Table 4). The sensitivity of different PCR techniques can be influenced by the different assays used to rupture the robust oocyst wall, the presence of PCR inhibitors, and the PCR protocols/procedures. Different methods to achieve efficient rupture of the oocyst wall such as bead beating, ultrasound, and freeze-and-thaw have been discussed [9], and many of the PCR-based studies reviewed here described the use of freeze-and-thaw or bead beating prior to DNA extraction that may increase analytical sensitivity [9]. In addition, the inclusion of an internal DNA amplification control (IAC) is recommended [9], as the presence of PCR inhibitors has been reported in soil, water (e.g., organic material), fresh produce, and bivalves, and IAC is mandatory for the detection of foodborne pathogens according to ISO 22174: 2005. It was rare for studies to report the use of an IAC (soil: [12,81], water: [109], fresh produce: [19,94], bivalves: [139]). Instead, some studies used commercial kits that included an appropriate PCR inhibitor removal step [53] or bovine serum albumin (BSA) [50,63,66,69,72,97,98,109], but the inhibition problem was not always solved.

DNA amplification methods (conventional PCR, nested PCR, qPCR, LAMP) targeted either B1, SAG1, 18sRNA, ITS-1, MIC3, GRA6, and 529RE markers [9]. The most commonly employed marker was B1 (in water, fresh produce, and bivalves) followed by 529RE (in soil), and a combination of both. This finding can be easily explained by the fact that sensitivity is increased when targeting multi-copy loci (B1, 529 RE, and ITS1) compared with single-copy gene targets (e.g., SAG1 and GRA6), as shown previously [22]. These PCR methods display high sensitivity but might lack in specificity as previously evidenced [22,166]. In fact, qPCR targeting the B1 gene and 529RE without probes cross-reacted with Sarcocystidae members [167]. Thus, powerful discrimination techniques are necessary to avoid false positive results and confirm species identity [22,37,121]. In this regard, the use of TaqMan probes in qPCR guarantees high specificity [9]. Alternative methods should be also taken into consideration. Amplicon sequencing and Restriction Fragment Length Polymorphism (RFLP) analyses have been used in some of the studies to confirm positive results (Table 1, Table 2, Table 3 and Table 4, Supplementary Tables S2–S5).

*Toxoplasma gondii* genotyping could help not only to confirm results but also to identify circulating genotypes. Genotyping tools (PCR-RFLP, microsatellite typing, PCR sequencing) were applied for this purpose in some studies, but in some instances, they were unsuccessful [19,119] or results were not reliable (e.g., based on a single marker [168]). The low oocyst burden observed in the environmental matrices (Supplementary Tables S2–S5) could limit the success of typing methods [169,170]. Currently, it is accepted that if samples are not fully characterized at the genotype level (https://toxodb.org/, accessed on 1 January 2022), the information gathered is not reliable enough for drawing robust conclusions [171,172]. Indeed, literature reviews have shown the low reliability of molecular data from environmental samples [8,172], since an unexpectedly high proportion of genotype I and non-canonical strains have been reported, which contrasts with the findings in samples from other sources such as domestic animals and humans from the same areas [172]. Therefore, additional efforts should be invested into unraveling the genotypes circulating in environmental matrices following an accurate and commonly accepted approach.

Finally, parasite quantification was not routinely carried out and was only estimated in a few studies conducted on soil, fresh produce, and bivalves. The limited data reported on parasite quantification were variable, as parasite load was referred to as the number of oocysts per g, per sample, per mL or µL, per DNA volume, or tachyzoite-equivalent copies. The quantity of *T. gondii* oocysts in soil varied from 11 to 2275 oocysts per mL [57] and 8 to 478 oocysts per 30 g of sample [81]. In fresh produce, the ranges were 1.31–900 oocysts per g of sample [94], 62–554 oocysts per g of vegetable matter [120], 0.6–179.9 oocysts (mean of 23.5 ± 12.1 oocysts per g) [37], <10–20 oocysts per sample (mean of three oocysts per sample) [38], and 0.3–27,640 oocysts per sample [19]. On the other hand, in bivalves, it ranged from 6 to 30 oocysts per sample [141] or per 5 µL of DNA [102], 1250 to 77,500 oocysts per sample (x = 24,694, SEM = 14,254.5) [140], 0.001 to 219 copies per µL of DNA [55], and 0.14 to 1.18 copies per g [137]. Means of 40–546 tachyzoite equivalents per mL were reported [139], as were Ct means of 39.1–40.7, which were equivalent to 0.1–1.4 oocysts [136]. However, the effect of matrix, as well as the effect of the performance of the reagents used and the lack of validation of the procedures, make the quantification questionable. Despite these variable results, parasite load was occasionally very high in the three matrices. Ideally, in this scenario, parasite viability should be estimated to define more clearly the risk that these matrices pose for humans.

Viability assays can be employed as detection methods and mouse bioassay has been suggested as a reference test for parasite detection, as mice are highly susceptible species to *T. gondii* infection [173]. In addition, bioassay methods can help to check the infectivity of the oocysts, and mouse bioassay has been widely employed for this purpose. The analysis of the literature evidenced that 15 of the studies attempted to isolate viable parasites mainly by bioassay in mice but also in pigs, chickens, and cats, and 11 obtained positive results (Table 1, Table 2, Table 3 and Table 4, Supplementary Tables S2–S5). Although standardized bioassay methods are needed [173], due to ethical concerns, new alternative techniques are required to discriminate between viable and inactivated oocysts. To date, there are some new proposals to estimate oocysts viability: propidium monoazide coupled with qPCR [29,31], staining with propidium iodide [31], reverse transcription quantitative PCR (RT-qPCR) [31,174], reverse transcription PCR (RT-PCR), excystation and dyes [175], and cell culture after oocysts excystation [174]. However, further studies are necessary to standardize these processes for different matrices and guarantee their correct performance.

## 4. Conclusions and Considerations for Future Research

The worldwide detection rates reported for the different environmental matrices covered in this systematic review, together with the published reports of confirmed human toxoplasmosis outbreaks due to contaminated soil, water, and fresh produce, provide evidence that environmental contamination with *T. gondii* oocysts poses a risk to public health. This is supported by the oocyst load/burden detected in different studies, which should not be underestimated given the fact that a single oocyst can cause infection, and that oocysts can persist in the environment for months or years, including in the marine environment [31]. Moreover, environmental oocyst contamination is a major source of infection for animal hosts, including animal hosts raised and hunted for human consumption [176,177,178]. This exemplifies that *T. gondii* is a pathogen that needs to be addressed using a One Health approach.

The timeline of the studies conducted on the different matrices is noteworthy. Fresh produce has been investigated only recently, and the number of studies is still limited. The timeline appears to be in line with the increasingly understood importance of other food- and waterborne zoonotic protists, in particular *Cryptosporidium* spp. Geographical gaps were also evident; many areas of the world of the world are significantly underrepresented in the studies: for example, sub-Saharan Africa. The overall detection rates of *T. gondii* were highly variable for each matrix, which can be partially explained by the different sampling strategies and methodologies employed. Differences in *T. gondii* detection in fresh produce have been attributed to variables such as the geographical location and methods used for parasite detection [9,33], which could also apply to other environmental matrices. Thus, it is important to consider both the sampling strategy and the methodology, as they can potentially influence parasite detection success and hamper comparisons between different studies. Regarding the sampling strategy, sampling areas, sample type, number, and mass or volume must be based on previous studies and available data such as reported toxoplasmosis cases in humans and animals, reported detection rates in environmental samples, expected detection rates, variability, and others. Regarding methodologies employed for environmental matrices, the most crucial steps to be considered are the spiking assays and the inclusion of an IAC to validate the recovery and detection methods. This would enable an estimation of analytical sensitivity and specificity and avoid false negatives results so that correct interpretation of the results would be guaranteed. Well-documented and standardized bioassays and genotyping methods will also help to determine the risk of exposure and how *T. gondii* circulates in the environment. Unfortunately, consensus guidelines have not yet been proposed by the scientific community. In the meantime, it would be advisable to include as much information as possible in publications, including details of experimental design and methodology.

More studies on *T. gondii* in environmental matrices are needed, and the focus should be on the gaps identified in this review. The impact of water contamination can be high, since its consumption is not limited by eating habits, as happens for vegetarians with meat-borne toxoplasmosis cases. In addition, water can contaminate soil, seafood, or fresh produce. Significant detection rates were found in surface water, in samples collected after a long treatment process, in irrigation/washing, and potable water. Moreover, the survival of oocysts in soil and the widespread consumption of minimally processed fresh produce and bivalves support the recommendation that *T. gondii*, as well as other cyst/oocyst forming protist parasites, should be included in regular food and water quality control guidelines within the food sector. Meanwhile, basic measures should be adopted by consumers such as washing of hands after handling soil or cat feces, washing fresh produce with clean water regardless of product presentation, and proper cooking of bivalves.

**Figure 3:**
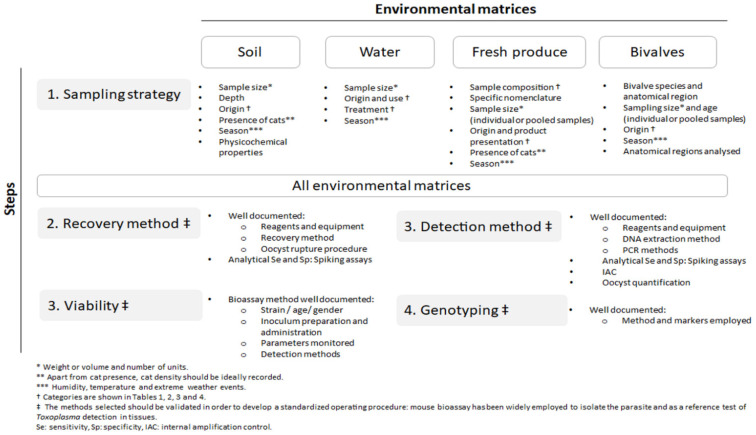
Workflow and key considerations for standard sampling strategies and detection methods for *Toxoplasma gondii* in environmental matrices.

Altogether, the relative contribution of different environmental matrices as *T. gondii* sources of infection to humans and animals remains unknown. Baseline data for risk assessment are limited and challenging to compare, since results may be influenced by sampling and methodological variables. Moreover, risk factors have not been adequately addressed in the context of the whole food chain including agricultural production and processing (incorporating soil, water, fresh produce including RTE products, and bivalves) given the limited and heterogeneous literature published. As an example, future work should investigate oocyst detection at the different steps of the RTE production workflow to implement mitigation strategies that might also help to avoid contamination with a wide variety of protozoa, helminths, fungi, and insects [179] and reduce infection risk for humans. Surveillance studies should ideally be accompanied by viability and genotyping assays to accurately determine the potential risk for consumers and enable tracing the sources. In general, all gaps identified evidenced the need to implement standardized procedures that could help to establish an ISO method and harmonize future studies focusing on environmental matrices. In Figure 3, we summarize the key aspects that should be considered when designing and implementing a study investigating *T. gondii* contamination of environmental matrices. As a minimum, these aspects should be explicitly addressed when reporting on the outcomes of such a study. Additional data to be considered could be extracted from systematic reviews and meta-analyses of risk factors for human infection with *T. gondii* (e.g., [180,181,182]). Similarly, the present review could also help further meta-analyses of risk factors in humans to identify relevant data. We appreciate the challenge of designing an adequately powered study, taking into account the multiple factors we have highlighted that can influence oocyst detection in environmental matrices. However, through the implementation of well-designed studies in the future, it will be possible to assess the contribution of different environmental matrices as sources of *T. gondii* infection to humans and animals and provide appropriate advice to policy makers, food producers, and consumers.

## Figures and Tables

**Table 5:** Subgroup analysis for comparison of the occurrence of *Toxoplasma gondii* oocysts detected by molecular methods in each matrix.

Matrix	No. of Studies Included	Pooled Detection Rates (95% CI)	Heterogeneity Test				Egger’s Test	
			I^2^ (%)	*Q (X* ^2^ *)*	Q/df	Q-p (*P)*	*t*	*p*
Soil	25	17.3 (11.0–23.7)	99.3	3388.03	24	<0.001	1.08	0.292
Water	28 ^a^	9.2 (6.3–12.0)	85.4	205.09	23	<0.001	2.33	0.030
Fresh produce	8 ^b^	5.2 (1.7–8.8)	78.2	36.76	8	<0.001	9.09	<0.001
Bivalve mollusks	10 ^c^	6.8 (4.4–9.2)	98.8	757.99	9	<0.001	2.82	0.030
Total	71 *	12.0 (10.0–14.0)	98.9	6679.21	74	<0.001	4.41	<0.001

I^2^, inverse variance index; Q, Cochran’s X^2^; Q-P *p*-value of Q-tests. * Few articles analyzed samples from more than one country. ^a^ Excluded: [59,89] (the number of positive samples was not specified). ^b^ Excluded: [60,78,92,119,120,121] (analyzed pooled samples). ^c^ Excluded: [54,102,117,130,131,132,136,138,139,140,141] (analyzed pooled samples).

## Data Availability

The present review has not been registered. All data collected and systematically searched are available in the Supplementary Tables provided.

## Supplementary Materials

The following are available online at https://www.mdpi.com/article/10.3390/microorganisms10030517/s1, Supplementary Figures S1–S4 (Forest plots for each matrix), Supplementary Figure S5 (Funnel plots for each matrix) and Supplementary Tables S1–S5: Excel file with the list of papers selected from each database and combination of search terms (Supplementary Table S1) and detailed data extracted from articles on soil (Supplementary Table S2), water (Supplementary Table S3), fresh produce (Supplementary Table S4), and bivalve mollusks (Supplementary Table S5).
